# Role of TRPC6 in kidney damage after acute ischemic kidney injury

**DOI:** 10.1038/s41598-022-06703-9

**Published:** 2022-02-22

**Authors:** Zhihuang Zheng, Dmitry Tsvetkov, Theda Ulrike Patricia Bartolomaeus, Cem Erdogan, Ute Krügel, Johanna Schleifenbaum, Michael Schaefer, Bernd Nürnberg, Xiaoning Chai, Friedrich-Alexander Ludwig, Gabriele N’diaye, May-Britt Köhler, Kaiyin Wu, Maik Gollasch, Lajos Markó

**Affiliations:** 1 Department of Nephrology/Intensive Care, Charité – Universitätsmedizin Berlin, Berlin, Germany; 2 Experimental and Clinical Research Center (ECRC), Max Delbrück Center for Molecular Medicine in the Helmholtz Association, Charité Universitätsmedizin, Berlin, Germany; 3 Department of Nephrology, Shanghai General Hospital, Shanghai Jiaotong University School of Medicine, Shanghai, China; 4 Department of Geriatrics, University of Greifswald, University District Hospital Wolgast, Greifswald, Germany; 5 Institute of Vegetative Physiology, Charité—Universitätsmedizin Berlin, Berlin, Germany; 6 Rudolf Boehm Institute for Pharmacology and Toxicology, Leipzig University, Leipzig, Germany; 7 Department of Pharmacology, Experimental Therapy and Toxicology and Interfaculty Center of Pharmacogenomics and Drug Research, University of Tübingen, Tübingen, Germany; 8 Department of Neuroradiopharmaceuticals, Institute of Radiopharmaceutical Cancer Research, Helmholtz-Zentrum Dresden-Rossendorf, Leipzig, Germany; 9 Department of Pathology, Charité—Universitätsmedizin Berlin, Berlin, Germany; 10 DZHK (German Centre for Cardiovascular Research), Partner Site Berlin, Berlin, Germany; 11 Berlin Institute of Health at Charité—Universitätsmedizin Berlin, Berlin, Germany; 12 Charité—Universitätsmedizin Berlin, Freie Universität Berlin, Humboldt-Universität zu Berlin, Berlin, Germany

**Keywords:** Kidney diseases, Nephrology, Acute kidney injury, Kidney

## Abstract

Transient receptor potential channel subfamily C, member 6 (TRPC6), a non-selective cation channel that controls influx of Ca^2+^ and other monovalent cations into cells, is widely expressed in the kidney. *TRPC6* gene variations have been linked to chronic kidney disease but its role in acute kidney injury (AKI) is unknown. Here we aimed to investigate the putative role of TRPC6 channels in AKI. We used *Trpc6*^*−/−*^ mice and pharmacological blockade (SH045 and BI-749327), to evaluate short-term AKI outcomes. Here, we demonstrate that neither *Trpc6* deficiency nor pharmacological inhibition of TRPC6 influences the short-term outcomes of AKI. Serum markers, renal expression of epithelial damage markers, tubular injury, and renal inflammatory response assessed by the histological analysis were similar in wild-type mice compared to *Trpc6*^*−/−*^ mice as well as in vehicle-treated versus SH045- or BI-749327-treated mice. In addition, we also found no effect of TRPC6 modulation on renal arterial myogenic tone by using blockers to perfuse isolated kidneys. Therefore, we conclude that TRPC6 does not play a role in the acute phase of AKI. Our results may have clinical implications for safety and health of humans with *TRPC6* gene variations, with respect to mutated TRPC6 channels in the response of the kidney to acute ischemic stimuli.

## Introduction

Transient receptor potential (TRP) channels are a group of ion channels located mostly on the plasma membrane of numerous cell types with a relatively large non-selective permeability to cations^1,2^. Mammalian TRP channel family comprises 28 members, which share some structural similarity with each other^2,3^. Transient receptor potential canonical or classical 6 (TRPC6) are non-selective Ca^2+^ permeable cation channels expressed in renal tissue including glomerular podocytes, mesangial cells, endothelial cells, tubulointerstitial vascular and epithelial cells, as well as in renal blood vessels^4^. Ca^2+^ influx through TRPC6 maintains the integrity of glomerular filtration barrier by interacting with nephrin, podocin, CD2-associated protein, and α-actinin-4 directly or indirectly^5^. Mutations in *TRPC6* lead to familial forms of focal segmental glomerulosclerosis (FSGS) and to end stage kidney disease^6,7^. Of note, TRPC6 dysregulation is also linked to progression of acquired forms of proteinuric kidney disease^8,9^. As a result of the TRPC6 activation, intracellular Ca^2+^ concentration in the podocyte increases and ultimately causes programmed cell death leading to progressive kidney failure. Recent evidence strongly suggests that TRPC6 also contributes to renal fibrosis and immune cell infiltration in the unilateral ureteral obstruction mouse model of progressive renal interstitial fibrosis^9,10^.

Initiation of renal fibrosis is often caused by acute kidney injury (AKI), an increasingly common complication occurring in critically ill patients with high morbidity and mortality^11^. The outcome of AKI has been primarily linked to the severity of tubular damage. In fact, most theories consider renal tubular cells as the main culprit in AKI. Ischemia/reperfusion injury (IRI)-induced epithelial damage—particularly in the outer medulla-, tubular obstruction, Ca^2+^ overload, loss of cytoskeletal integrity, and loss of cell–matrix adhesion are main consequences of ischemia and initiators of subsequent fibrosis^12,13^. Recent data show that in vivo TRPC6 inhibition by BI-749327 ameliorates renal fibrosis in the unilateral obstructive nephropathy (UUO) model^14^, where the primary feature is tubular injury as a result of obstructed urine flow. Ischemia is frequently involved in AKI and correlates with oxidative stress and inflammation^15,16^. Reactive oxygen species (ROS) produced by NADPH oxidases (NOX) contribute to activation of TRPC6 channels^17,18^. Moreover, TRPC6 is emerging as a functional element to control calcium currents in immune cells, thereby regulating transendothelial migration, chemotaxis, phagocytosis, and cytokine release^19,20^. In a recent study, Shen et al*.* found that silencing TRPC6 could prevent necroptosis of renal tubular epithelial cells upon IRI^21^. These findings indicate that inhibition of TRPC6 may protect the kidney from IRI making it a promising target to ameliorate AKI. This is of special interest and of potential clinical relevance as novel chemical substances selectively acting on TRPC6 have been recently developed for in vivo experiments. In particular, the diterpene (+)-larixol and its derivative SH045, as well as BI-749327 which is another known TRPC6 blocker, have been developed for selective inhibition of TRPC6 and translational treatment of TRPC6 channelopathies^14,22,23^.

To the best of our knowledge, no studies investigated the potential role of TRPC6 channels in AKI. Therefore, we tested the hypothesis that TRPC6 inhibition is renoprotective in AKI. We induced AKI by ischemia reperfusion injury in wild-type (WT) and *Trpc6*^*−/−*^ mice. Furthermore, we used pharmacological blockers (SH045 and BI-749327) in vivo and ex vivo to verify findings from the genetical model and to evaluate effects of TRPC6 inhibition in IRI-induced AKI and intrarenal regulation of blood flow. Serum markers, renal damage marker expression, histological analysis of renal tissue damage and cellular infiltration were performed. We conclude that neither lacking nor pharmacological inhibition of TRPC6 ameliorates short-term outcomes of AKI.

## Results

### The impact of *Trpc6* deficiency on renal damage after renal IRI

**Figure 1:**
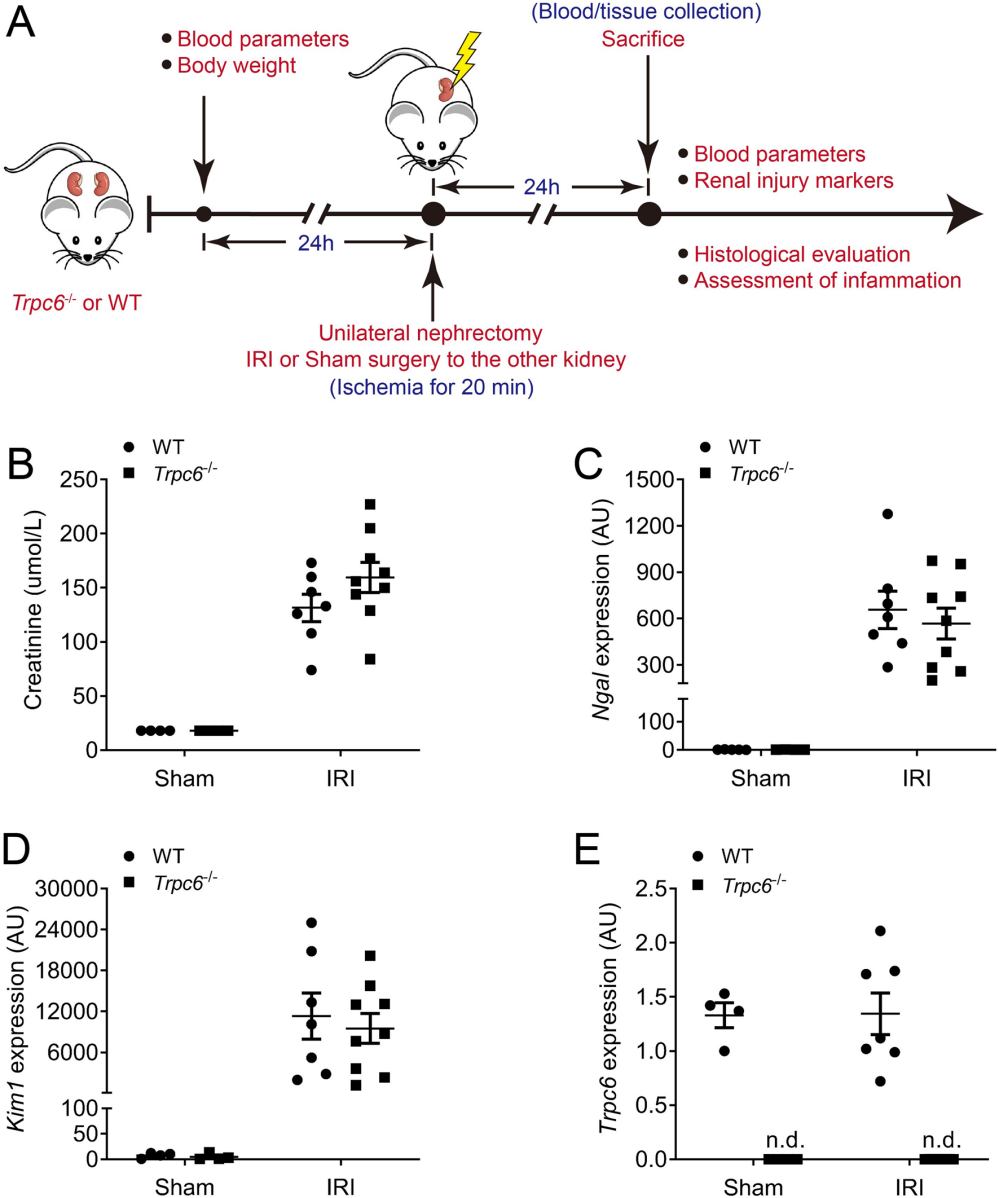
Effect of *Trpc6* deficiency on IRI-induced acute kidney injury (AKI). (**A**) Experimental design of ischemia reperfusion-induced AKI. (**B**) Serum creatinine levels in the experimental groups (Sham WT and *Trpc6*^*−/−*^ n = 4 each, IRI WT n = 7, and *Trpc6*^*−/−*^ n = 9, respectively). (**C**) Renal mRNA levels of kidney injury marker neutrophil gelatinase-associated lipocalin (*Ngal*), (**D**) kidney injury molecule 1 (*Kim1*) and (**E**) *Trpc6* (Sham WT and *Trpc6*^*−/−*^ n = 4 each, IRI WT n = 7, and *Trpc6*^*−/−*^ n = 9, respectively). Please note that at baseline all serum creatinine levels were below the measurement limit (18 µmol/L). Two-way ANOVA followed by Sidak’s multiple comparisons post hoc test. *AU* arbitrary units, *n.d.* not detected.

To examine a possible role of *Trpc6* deficiency in AKI, we performed comparative in vivo studies using *Trpc6*^*−/−*^ and WT mice (Fig. 1A). Twenty-four hours after IRI WT mice showed similar serum creatinine level compared to *Trpc6*^*−/−*^ mice (*P* = 0.18; Fig. 1B). Renal expression of tubular damage marker neutrophil gelatinase-associated lipocalin (*Ngal*) and kidney injury molecule 1 (*Kim1*) showed no difference between WT and *Trpc6*^*−/−*^ mice (Fig. 1C,D). Additionally, *Trpc6* expression in kidneys after IRI was not altered compared to the sham kidneys (Fig. 1E). At baseline *Trpc6*^*−/−*^ mice had similar blood parameters compared to WT mice (except higher but still normal concentrations of sodium and ionized Ca^2+^ (Supplementary Table S2A). After IRI, WT and *Trpc6* deficient mice developed similar hyperkalaemia and similar high serum levels of creatinine and urea nitrogen (Supplementary Table S2B). In contrast, concentrations of sodium and chloride were lower in *Trpc6*^*−/−*^ mice (Supplementary Table S2B).

### The impact of *Trpc6* deficiency on renal histopathology after IRI

**Figure 2:**
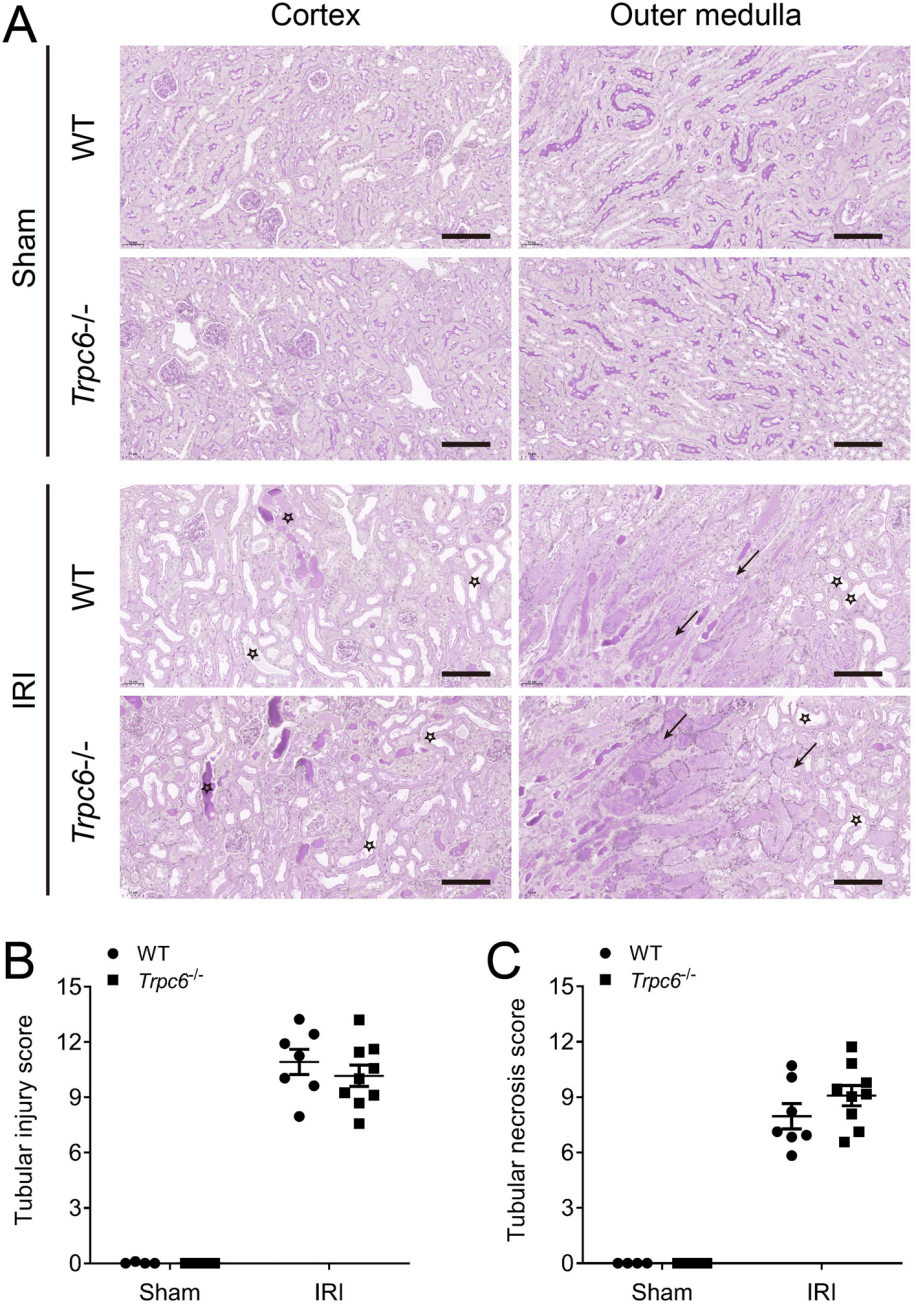
Effect of *Trpc6* deficiency on kidney histopathology after renal IRI–induced AKI. (**A**) Representative cortical or outer medullary images of IRI-injured kidneys isolated from *Trpc6*^*−/−*^ and WT mice (magnification: 200 ×). Kidneys sections were stained with periodic acid-Schiff staining (PAS). Arrows indicate tubular necrosis. Stars indicate tubular injury. Scale bars are 100 µm. (**B**) Semi-quantification of cortical tubular injury. (**C**) Semi-quantification of outer medullary tubular necrosis. Data expressed as means ± SEM (Sham WT and *Trpc6*^*−/−*^ n = 4 each, IRI WT n = 7, and *Trpc6*^*−/−*^ n = 9, respectively). Two-way ANOVA followed by Sidak’s multiple comparisons post hoc test.

To assess the degree of tubular damage in ischemic AKI kidney sections were analyzed by Periodic Acid-Schiff (PAS) staining (Fig. 2A). IRI induced severe tubular damage and necrosis such as tubular epithelial swelling, loss of brush border, luminal dilatation with simplification of the epithelium, patchy loss of tubular epithelial cells with resultant gaps and exposure of denuded basement membrane, as well as obliterated tubular hyaline and/or granular casts (Fig. 2A). Analysis of the kidney sections revealed no differences in tubular injury score and tubular necrosis score between *Trpc6*^*−/−*^ and WT mice (Fig. 2B,C).

### The impact of *Trpc6* deficiency on cellular infiltration and on the expression of calcium-binding proteins in the kidneys after IRI

**Figure 3:**
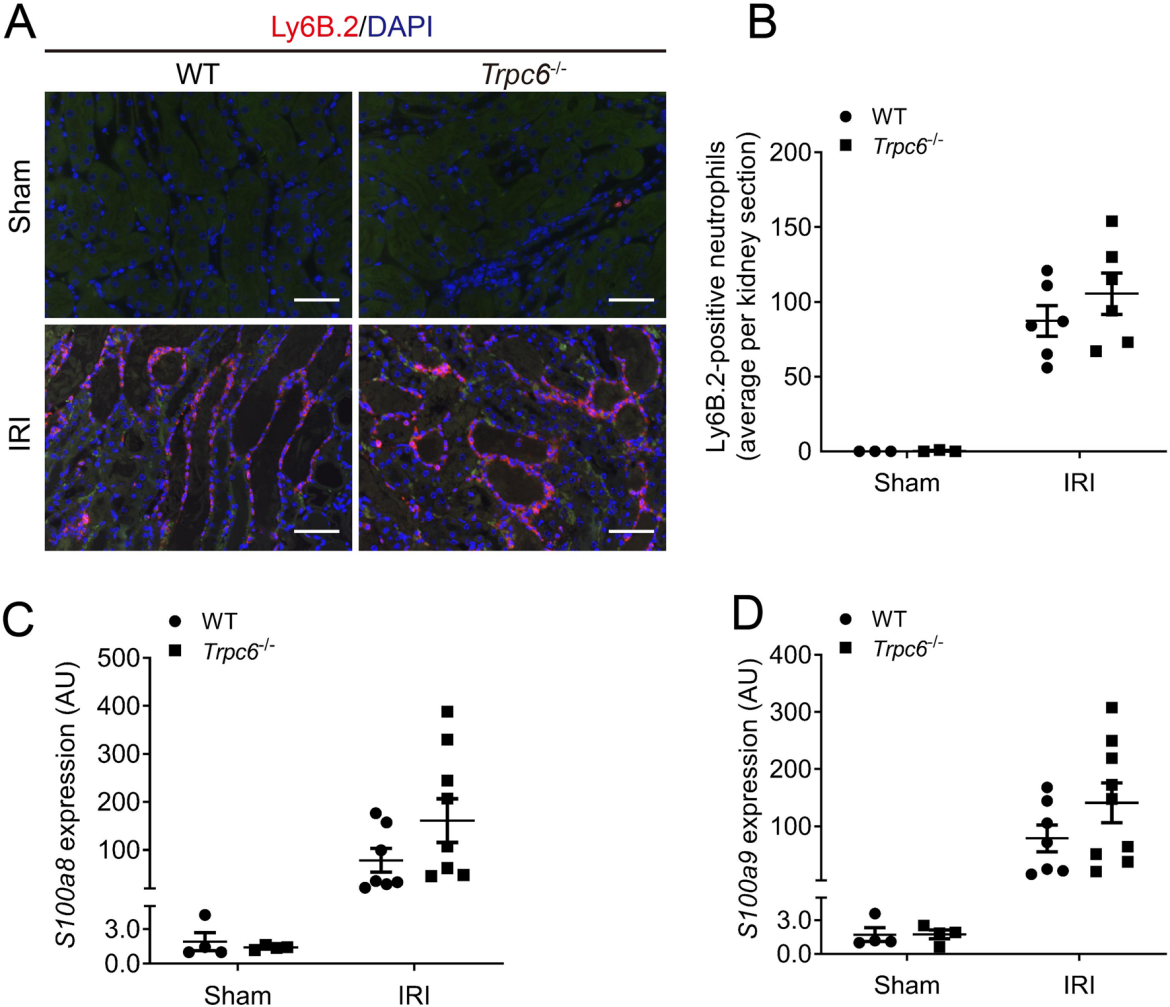
Effect of *Trpc6* deficiency on renal neutrophils infiltration and inflammatory markers in IRI–induced AKI. (**A**) Representative outer medullar images of IRI-injured kidneys. Kidneys were stained with Ly6B.2 (magnification: 400 ×). Scale bars are 100 µm. (**B**) The semi-quantification renal neutrophils infiltration (Sham WT and *Trpc6*^*−/−*^ n = 3 each, IRI WT n = 6, and *Trpc6*^*−/−*^ n = 6, respectively). (**C**) Renal mRNA levels of S100 calcium-binding protein A8 (*S100a8*). (**D**) Renal mRNA levels of S100 calcium-binding protein A9 (*S100a9*) (Sham WT and *Trpc6*^*−/−*^ n = 4 each, IRI WT n = 7, and *Trpc6*^*−/−*^ n = 9, respectively). Data expressed as means ± SEM. Two-way ANOVA followed by Sidak’s multiple comparisons post hoc test. *AU* arbitrary units.

Neutrophils infiltration after IRI-induced AKI contributes to inflammation and subsequent repair of injured kidneys^24,25^. Here, we determined neutrophils infiltration in renal IRI using immunofluorescence staining. Kidney sections were immunolabelled with the neutrophil marker Ly6B.2^26^. As shown in Fig. 3A, excessive Ly6B.2-positive neutrophils in renal interstitium were observed in mice underwent IRI (Fig. 3A). *Trpc6*^*−/−*^ mice showed similar amount of infiltratating Ly6B.2-positive cells after IRI as WT mice (Fig. 3B). Similarly, renal expression of neutrophil markers S100 calcium-binding protein A8 (*S100a8*) and S100 calcium-binding protein A9 (*S100a9*) were not different in IRI-damaged renal tissues of *Trpc6*^*−/−*^ and WT mice (Fig. 3C,D).

### Expression of inflammatory markers in WT and *Trpc6*^*−/−*^ kidneys

**Figure 4:**
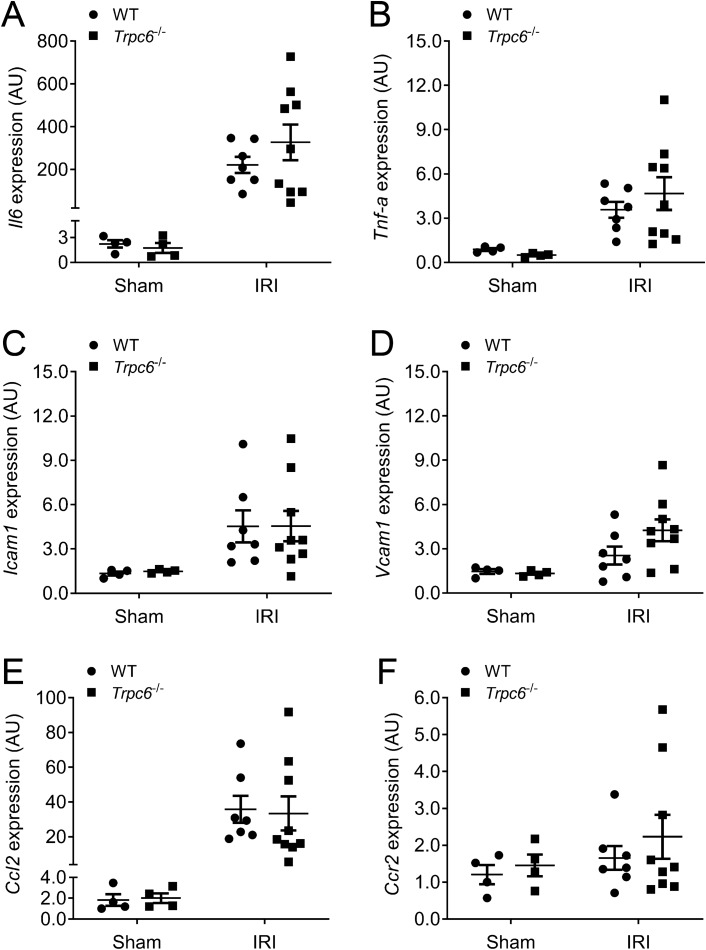
Effect of *Trpc6* deficiency on gene expression of pro-inflammatory cytokines and chemokines after renal IRI. (**A**) Renal mRNA levels of interleukin 6 (*Il6*) and (**B**) tumor necrosis factor-α (*Tnf-α*), (**C**) intercellular adhesion molecule 1 (*Icam1*) and (**D**) vascular cell adhesion protein 1 (*Vcam1*), (**E**) C–C motif chemokine 2 (*Ccl2*) and (**F**) C–C motif chemokine receptor 2 (*Ccr2*) (Sham WT and *Trpc6*^*−/−*^ n = 4 each, IRI WT n = 7, and *Trpc6*^*−/−*^ n = 9, respectively). Data expressed as means ± SEM. Two-way ANOVA followed by Sidak’s multiple comparisons post hoc test. *AU* arbitrary units.

Expression of inflammatory molecules is low in the normal kidney but is markedly increased under pathophysiological conditions such as IRI-induced AKI^27^. By using qPCR, we examined the mRNA expression of cell adhesion molecules and inflammatory markers involved in renal IRI. IRI led to increased mRNA expression of all determined markers (Fig. 4A–F), however *Trpc6*^*−/−*^ IRI kidneys displayed similar expression of interleukin 6 (*Il6*), tumor necrosis factor-alpha (*Tnf-a*), intercellular adhesion molecule 1 (*Icam1*), vascular cell adhesion molecule 1 (*Vcam1*) in comparison to WT IRI kidneys (Fig. 4A–D). In addition, renal expression of chemokines such as chemokine (C–C motif) ligand 2 (*Ccl2*) and chemokine (C–C motif) ligand 2 receptor (*Ccr2*), were similar between *Trpc6*^*−/−*^ and WT mice (Fig. 4E,F).

### The impact of Trpc6 pharmacological blockade on renal damage after renal IRI

**Figure 5:**
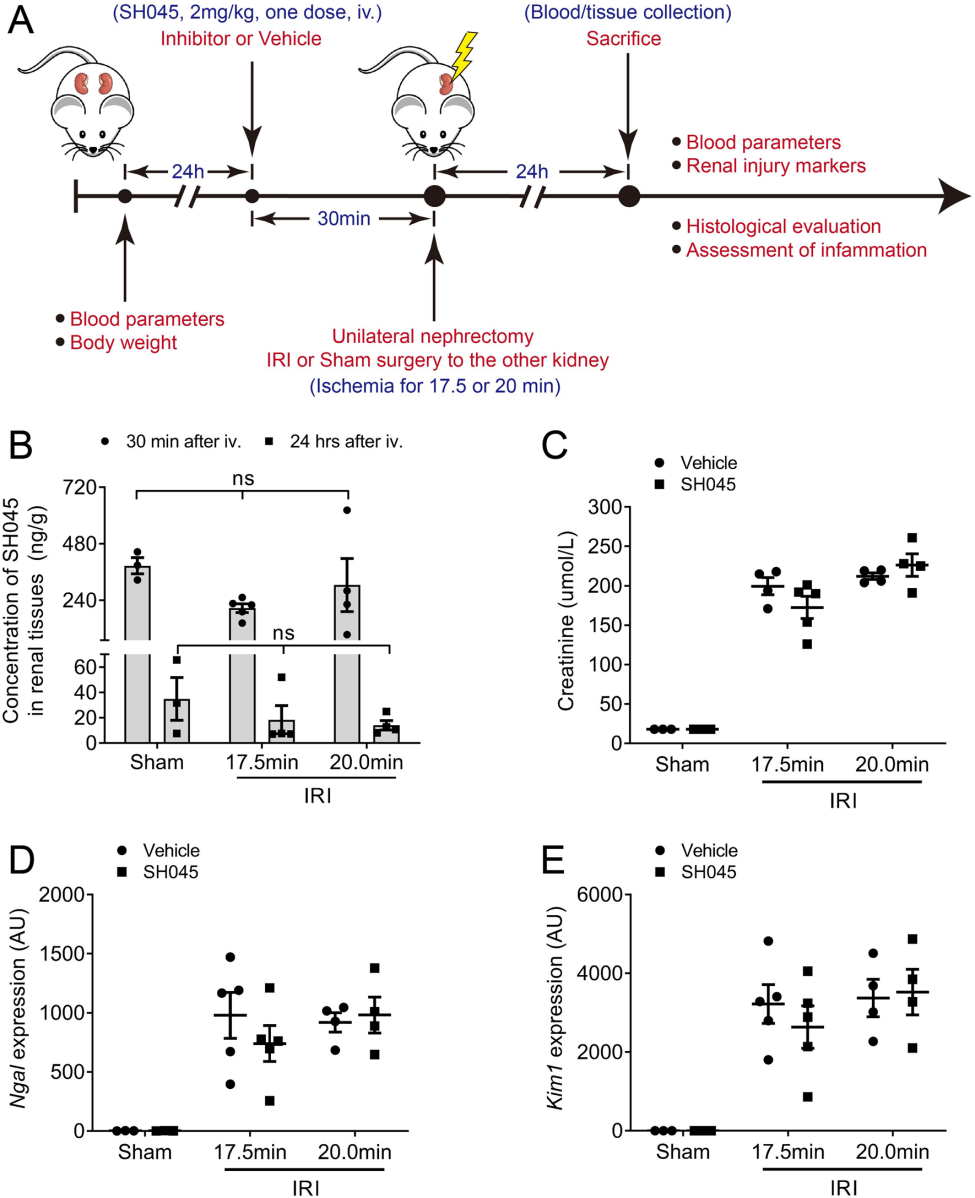
Effect of the TRPC6 inhibitor SH045 on IRI–induced acute kidney injury (AKI). (**A**) Experimental design of IRI–induced AKI. (**B**) Concentrations of SH045 in kidney tissue 30 min and 24 h after intravenous injection (Sham n = 3 each, 17.5 min-IRI in vehicle and SH045 n = 5 each, 20 min-IRI in vehicle and SH045 n = 4 each, respectively). (**C**) Serum creatinine levels (Sham n = 3 each, 17.5 min-IRI in vehicle and SH045 n = 5 each, 20 min-IRI in vehicle and SH045 n = 4 each, respectively). (**D**) Renal mRNA levels of kidney injury marker neutrophil gelatinase-associated lipocalin (*Ngal*) and (**E**) kidney injury molecule 1 (*Kim1*) (Sham n = 3 each, 17.5 min-IRI in vehicle and SH045 n = 5 each, 20 min-IRI in vehicle and SH045 n = 4 each, respectively). Please note that at baseline all serum creatinine levels were below the measurement limit (18 µmol/L). Two-way ANOVA followed by Sidak’s multiple comparisons post hoc test. *AU* arbitrary units. Data expressed as means ± SEM.

To circumvent possible bias due to the genetical approach and to examine effects of pharmacological blockade of Trpc6 on renal damage, we used SH045 (a recently developed drug with high affinity and strong subtype selectivity toward TRPC6) in vivo in the ischemic AKI mouse model using two different ischemia time (milder with 17.5 min and a stronger with 20 min) (Fig. 5A). Renal concentrations of SH045 in both 30 min and 24 h after intravenous injection were similar between sham and IRI groups (Fig. 5B). Consistent with AKI data from *Trpc6*^*−/−*^ and WT mice, pharmacological blockade of TRPC6 had no effects on serum creatinine levels after 17.5 min or 20 min IRI-induced AKI in comparison to vehicle injected WT IRI mice (*P* = 0.23 and *P* = 0.76, respectively, Fig. 5C). In addition, renal mRNA expression of *Ngal* and *Kim1* was similar in SH045-treated compared to vehicle-treated kidneys after IRI (Fig. 5D,E). Furthermore, SH045 did not affect blood parameters such as sodium, potassium, chloride, ionized calcium, carbon dioxide, glucose, urea nitrogen, hematocrit, hemoglobin, and the anion gap after IRI-induced AKI (Supplementary Table S3A–F). To further confirm the results of SH045, we used another TRPC6 blocker, BI-749327. In IRI-induced AKI mice, renal function parameters such as serum creatinine and renal expression of renal damage markers (*Ngal* and *Kim1*) were similar between vehicle- and BI-749327-treated mice (Supplementary Fig. S1A–D). Besides, no difference in other blood parameters was found between vehicle- and BI-749327-treated AKI mice (Supplementary Table S4A,B).

### The impact of Trpc6 pharmacological blockade on renal histopathology after IRI

**Figure 6:**
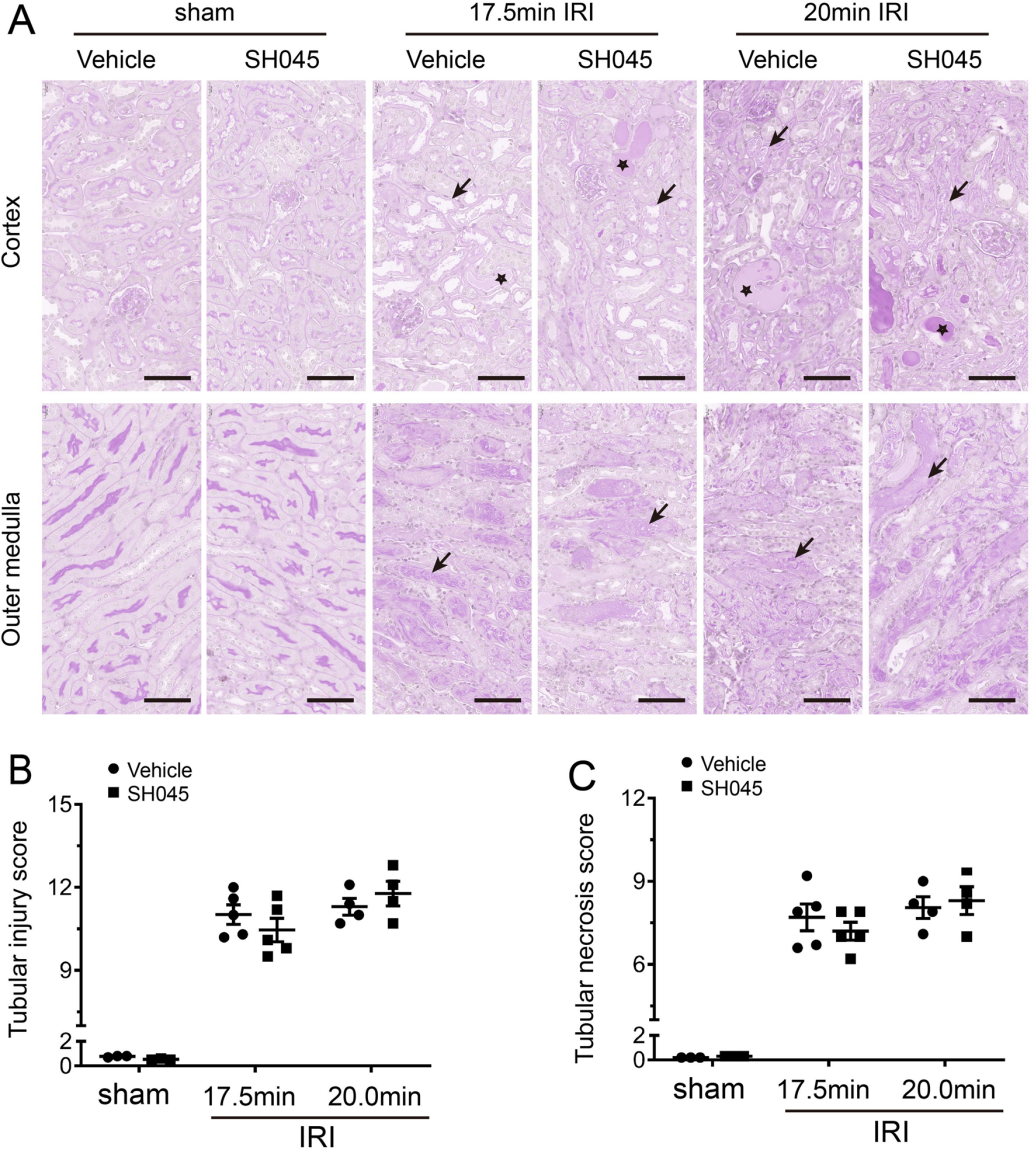
SH045 effects on kidney histopathology after renal ischemia reperfusion–induced AKI. (**A**) Representative cortical or outer medullary images of IRI-injured kidneys isolated from mice injected with vehicle or Trpc6 blocker SH045 (magnification: 400 ×). Kidneys sections/slices were stained with Periodic Acid-Schiff staining (PAS). Arrows indicate tubular injury. Stars indicate tubular casts. Scale bars are 100 µm. (**B**) The semi-quantification of cortical tubular injury. (**C**) The semi-quantification of outer medullary tubular necrosis. Data expressed as means ± SEM (Sham n = 3 each, 17.5 min-IRI in vehicle and SH045 n = 5 each, 20 min-IRI in vehicle and SH045 n = 4 each, respectively). Two-way ANOVA followed by Sidak’s multiple comparisons post hoc test.

Histological analyses and semi-quantitative scoring revealed no statistically significant differences in cortical tubular damage after renal 17.5 min- or 20 min-IRI in SH045 compared to vehicle-treated mice (Fig. 6A,B). Moreover, epithelial cells in the outer stripe of outer medulla, especially susceptible to ischemic injury, exhibited comparable level of tubular necrosis between SH045 treated and control mice after renal 17.5 min- or 20 min-IRI (Fig. 6A,C). Similarly, BI-749327 also did not influence tubular damage and necrosis in IRI (Supplementary Fig. S2A–C).

### The impact of Trpc6 pharmacological blockade on cellular infiltration and calcium-binding proteins on the kidneys after IRI

**Figure 7:**
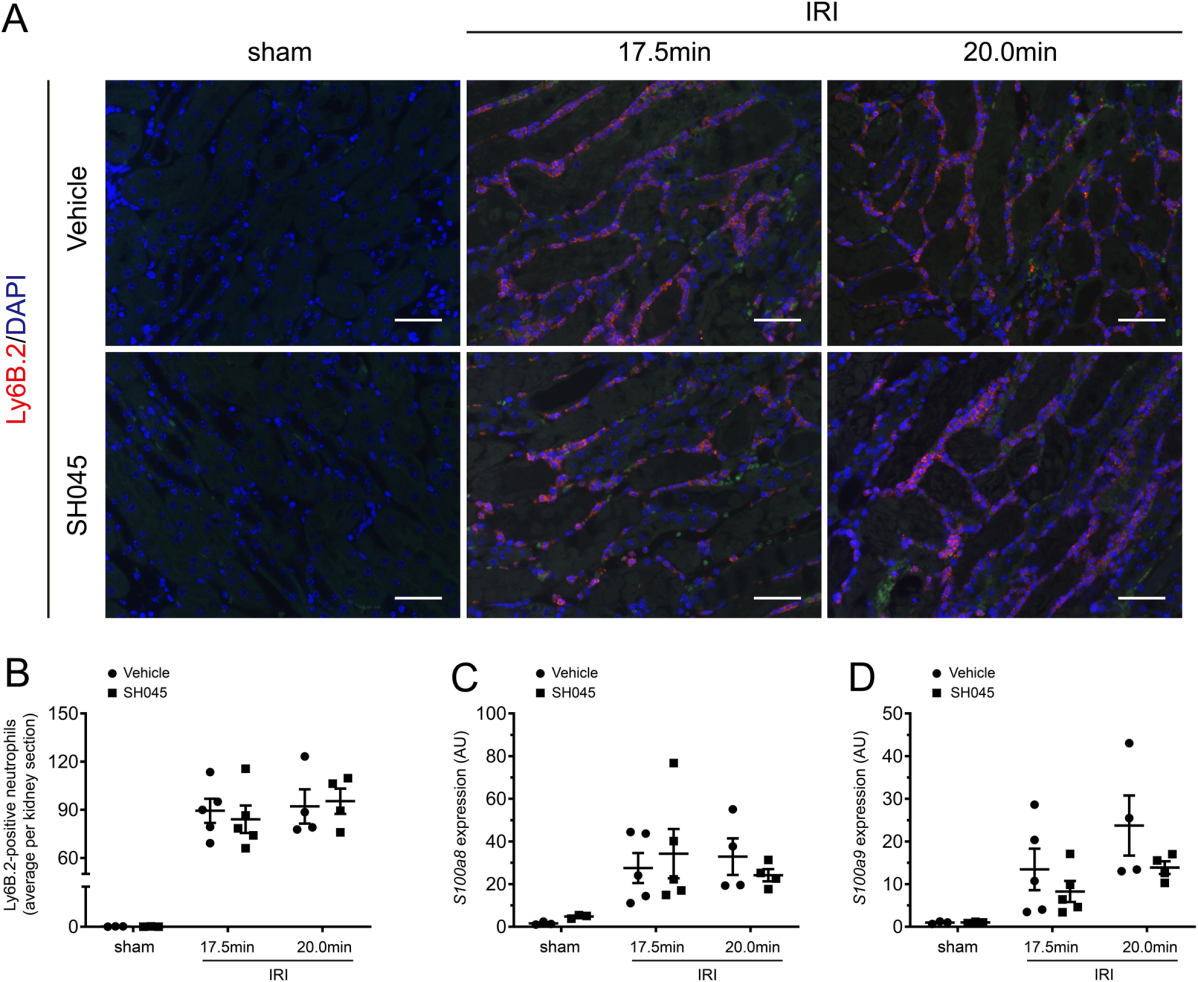
SH045 effects on renal neutrophils infiltration and inflammatory markers in ischemia reperfusion–induced AKI. (**A**) Representative outer medullar images of IRI-injured kidneys. Kidneys were stained with Ly6B.2 (magnification: 400 ×). Sham and IRI mice were treated with either vehicle or Trpc6 inhibitor (SH045). Scale bars are 100 µm. (**B**) The semi-quantification renal neutrophils infiltration. (**C**) Renal mRNA levels of S100 calcium-binding protein A8 (*S100a8*). (**D**) Renal mRNA levels of S100 calcium-binding protein A9 (*S100a9*). Data expressed as means ± SEM (Sham n = 3 each, 17.5 min-IRI in vehicle and SH045 n = 5 each, 20 min-IRI in vehicle and SH045 n = 4 each, respectively). Two-way ANOVA followed by Sidak’s multiple comparisons post hoc test. AU, arbitrary units.

Consistent with the results in AKI-induced *Trpc6*^*−/−*^ mice**,** SH045 treatment had no effect on renal infiltration of Ly6B.2-positive granulocytes after 17.5 min- or 20 min-IRI-induced AKI in comparison to vehicle-treated mice (Fig. 7A,B). In agreement with that, renal expression of *S100a8* and *S100a9* after 17.5 min- or 20 min-IRI is similar in mice treated with SH045 versus vehicle (Fig. 7C,D). Furthermore, similar results were also obtained in mice treated with BI-749327 compared to vehicle-treated mice (Supplementary Fig. S3G,H).

### The impact of Trpc6 pharmacological blockade on expression of renal cytokines and chemokine after IRI

**Figure 8:**
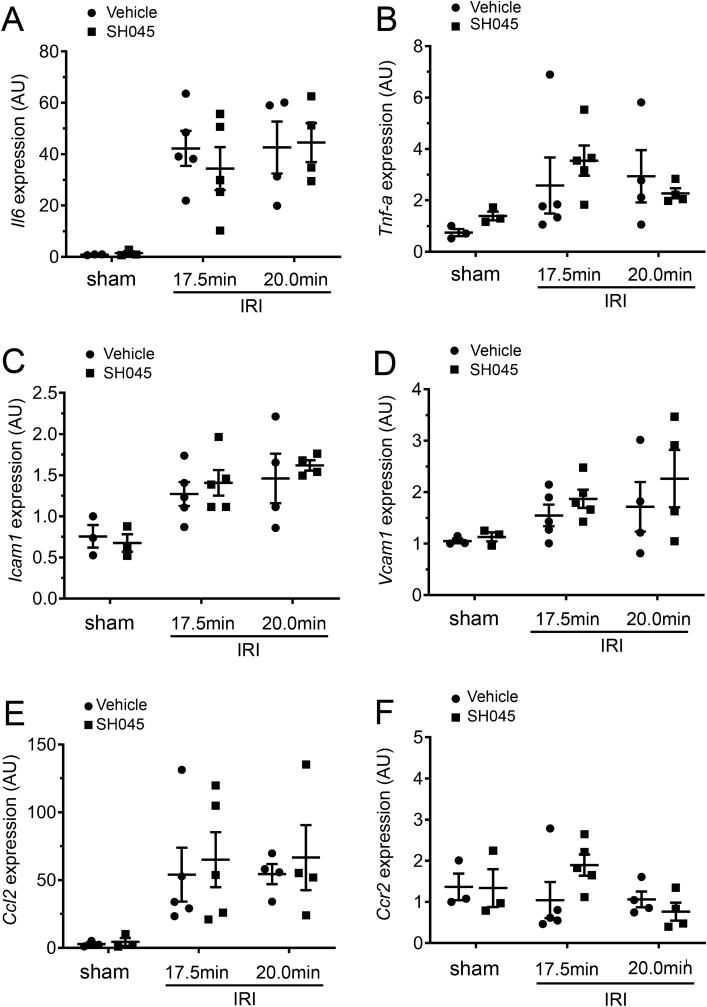
SH045 effects on gene expression of pro-inflammatory cytokines and chemokines after renal IRI. (**A**) Renal mRNA levels of interleukin 6 (*Il 6*), (**B**) tumor necrosis factor-α (*Tnf-α*), (**C**) intercellular adhesion molecule 1 (*Icam1*) and (**D**) vascular cell adhesion protein 1 (*Vcam1*). (**E**) Renal mRNA levels of C–C motif chemokine 2 (*Ccl2*) and (**F**) C–C motif chemokine receptor 2 (*Ccr2*). Data expressed as means ± SEM (Sham n = 3 each, 17.5 min-IRI in vehicle and SH045 n = 5 each, 20 min-IRI in vehicle and SH045 n = 4 each, respectively). Two-way ANOVA followed by Sidak’s multiple comparisons post hoc test. *AU* arbitrary units.

SH045-treated mice underwent 17.5 or 20 min IRI-induced AKI showed similar renal expression of *Il6*, *Tnf-α*, *Icam1*, *Vcam1*, *Ccl2*, and *Ccr2* in comparison to vehicle-treated mice with the same IRI (Fig. 8A–F). In addition, the mRNA levels of these inflammatory markers in BI-749327-treated AKI mice were equivalent to vehicle-treated AKI mice (Supplementary Fig. S3A–F).

### Impact of pharmacological modulation of Trpc6 on renal microcirculation

The renal microcirculation is emerging as a key player in AKI^28^. To explore whether modulation of TRPC6 has impact on renal microcirculation, we evaluated myogenic tone in the mouse renal circulation using the TRPC6 blockers SH045 and BI-749327, and the TRPC6 activator hyperforin^29^. Isolated kidneys perfused with SH045 or BI-749327 developed a similar decrease of perfusion pressure compared to control kidneys (Supplementary Fig. S4A–C,E). In addition, activation of TRPC6 using hyperforin resulted in similar spontaneous decrease of perfusion pressure compared to control kidneys (Supplementary Fig. S4A,D,E). Of note, there was also no difference in Angiotensin (Ang) II induced vasoconstrictions between kidneys perfused with SH045, BI-749327, or hyperforin versus control kidneys (Supplementary Fig. S4F). Together, these results indicate no effect of TRPC6 modulation on renal arterial myogenic tone.

## Discussion

In our previous study, we have shown that TRPC6 contributes to renal fibrosis and immune cell infiltration in a murine UUO model using *Trpc6*^*−/−*^ mice^9^. Renal IRI is a common cause of CKD. IRI is associated with calcium (Ca^2+^) overload, ROS production, and immune responses which has been reported as crucial factors of tubular injury in IRI-induced AKI^12,15,16^. As a Ca^2+^-permeable cationic channel, TRPC6 can be activated by reactive oxygen species (ROS) and mediate podocyte injury in glomerular diseases^17,18^. In addition, TRPC6 is involved in immune responses regulating transendothelial migration, chemotaxis, phagocytosis, and cytokine release^19,20^. A recent study using pharmacological blockage of TRPC6 in mice demonstrated that decreased Ca^2+^ entry due to TRPC6 contributed to reducing astrocytic apoptosis, cytotoxicity and inflammatory responses in cerebral ischemic/reperfusion insult^30^. Given that, in the present study we hypothesized that TRPC6 inhibition may also exhibit nephroprotective effects in AKI. To test this hypothesis, we applied the IRI experimental model on *Trpc6*^*−/−*^ mice and employed a pharmacological approach of in vivo TRPC6 inhibition by SH045 and BI-749327 in wild-type (WT) mice.

In our study, *Trpc6*^*−/−*^ mice subjected to acute IRI showed no difference in renal function or tubular damage when compared to WT mice which underwent IRI. The *Trpc6*^*−/−*^ mouse model was established as a homozygous colony, therefore we used C57BL/6 J mice as control^9^. Given this limitation of our experimental approach and to account for possible confounding genomic and non-genomic effects of other TRPC channels caused by global loss of TRPC6^31^, we performed also studies using two different TRPC6 blockers SH045 and BI-749327 in C57BL/6 J WT mice to evaluate the role of TRPC6 in short-term AKI outcomes. Of note, TRPC6 and TRPC2, TRPC3, TRPC7 can form heteromeric channels with regulatory properties that are different from those of the homomeric TRPC channels^32,33^. Pharmacological inhibition allows high selectivity for inhibition of homomeric TRPC6 channels than for heteromeric channels in AKI, particularly compared to targeting TRPC6 using *Trpc6*^*−/−*^ animals. Thus, using the pharmacological approach can precisely allow investigating the specific effects of TRPC6 homomeric channels on AKI. We used SH045 and BI-749327, and performed studies with two different ischemic times in order to have a model for mild (17.5 min) and moderate (20 min) renal damage. These two periods of reversible ischemia were based on our earlier IRI studies with mice^34–36^. However, we still found no differences with respect to serum creatinine or tubular damage between mice undergoing IRI treated with TRPC6 blocker *vs.* vehicle in both models.

Previously, we demonstrated that inhibition of tubular epithelial NF-κB activity can ameliorate IRI-induced AKI in our experimental settings^36^. Thus, our experimental conditions enable to observe beneficial effects of therapeutic interventions, i.e. kidney injury potentially can be ameliorated in our AKI mouse model. According to the reported IC_50_-values for SH045 of 6 to 60 nM^37^ and the pharmacokinetic analysis by non-compartment modeling^38^, SH045 can be considered as pharmacologically effective at tissue concentrations equal or higher than 22 ng/g. To confirm the drug is distributed in the kidney within the expected time, we measured the renal concentration of SH045 by using LC–MS/MS assay. As shown in Fig. 5B, the concentration of SH045 in renal tissue after 30 min post-injection (284.56 ng/g ± 144.76) was much higher than 22 ng/g. In case of BI-749327, Lin et al., reported that in concentrations of 10–30 mg/kg BI-749327 has renal protective effects in the UUO mouse model^14^. Therefore, the concentrations of pharmacological blockers used in this study were appropriate to inhibit the TRPC6 channels in vivo in our experiments.

It is now well established that inflammation plays an important role in AKI^39–42^. Shortly after endothelial or tubular epithelial cell damage, activation of resident renal inflammatory cells occurs. It leads to overproduction of cytokines, followed by recruitment and subsequent infiltration with different leukocytes subsets^43,44^. TRPC6 is expressed in a wide range of inflammatory and vascular cell types, including neutrophils, lymphocytes and endothelium^45^. During the acute phase response, TRPC6 plays a crucial role in neutrophil mobilization as it enhances chemotactic responses through increasing intracellular Ca^2+^ concentration and promoting actin-based cytoskeleton remodeling^20,46^. Indeed, after renal IRI, we found a notable accumulation of neutrophils in the region of outer medulla. However, the infiltration of neutrophils in IRI *Trpc6*^*−/−*^ kidney was equivalent to IRI WT mice as well as in SH045-treated AKI mice compared to vehicle-treated AKI mice. Accordingly, inflammatory response including cytokines and chemokine overexpression was also similar in AKI mice with or without Trpc6 intervention. In addition, *Trpc6* was normally expressed in kidney tissue 24 h after IRI. A recent study using single-cell RNA sequencing in clusters of inflammatory cells during AKI confirms a similar renal expression of *Trpc6* mRNA in AKI versus control^47^. S100a8 and S100a9 are Ca^2+^ binding proteins belonging to the S100 family, which are constitutively expressed in neutrophils and monocytes as a Ca^2+^ sensor, participating in cytoskeleton rearrangement and arachidonic acid metabolism^48,49^. These two molecules modulate inflammatory response by stimulating recruitment of neutrophils and cytokine secretion^49^. We hypothesized that blocking intracellular influx of Ca^2+^ in neutrophils through genetical or pharmacological TRPC6 inhibition might affect the S100a8/9-mediated inflammation. However, we detected comparable renal expression of *S100a8/9* after AKI in WT and *Trpc6*^*−/−*^ mice or in TRPC6 blockers-treated kidneys after AKI compared to respective control kidneys. Taken together, our data suggest no impact of TRPC6 to renal inflammatory reaction in the ischemic AKI.

Of note, TRPC6 is also expressed in tubular epithelial cells and plays an important role in nephron physiology^50^. Although one study showed functional importance of TRPC6 in autophagy regulation in vitro using proximal tubular cells of *Trpc6* deficient mice and TRPC6 overexpressing HK-2 cells^50^, our data argue against protective effects of TRPC6 in the tubular damage of AKI in vivo*.* After AKI, interstitial fibrosis formation is an important part of renal repair, yet excess leads to AKI-to-CKD transition. Wu et al., and our previous study showed that inhibition of TRPC6 function either by genetic deletion ameliorates renal interstitial fibrosis^10^. Besides, another study further demonstrated an obligate function for TRPC6 in promoting fibroblast transdifferentiation^51^. Therefore, inhibition of TRPC6 may have effects on renal fibrogenesis during AKI-to-CKD transition, and the beneficial effects of TRPC6 inhibition seen in the UUO model most likely involve fibroblast activation and transdifferentiation. Since its differentiation is an expected long-term outcome of AKI^52^, this might explain the lack of *Trpc6*^*−/−*^ effects on the short-term outcome of AKI in present study. In addition, renal microvascular dysfunction is considered playing critical role in acute kidney injury^53^. Due to unchanged myogenic tone after TRPC6 modulation in vascular smooth muscle cells using isolated perfused kidneys, it is unlikely that a vascular component represents a protective mechanism of renal TRPC6 inhibition.

In summary, the present study shows that TRPC6 inhibition has no effects on short-term outcome of AKI. Our results improve the understanding of the role of TRPC6 role in kidney diseases. Future studies should investigate if TRPC6 could have a protective role on long-term outcome of AKI. Our results may have clinical implications for safety and long-term outcome of humans with *TRPC6* gene variations leading to familial forms of FSGS, with respect to the response of the kidney to acute ischemic stimuli.

## Materials and methods

### Animals

Male *Trpc6*^*−/−*^ mice (n = 10, homozygosity in a mix 129 Sv:C57BL/6J background) and wild–type (WT, C57BL/6J) control mice (n = 9) were used. *Trpc6*^*−/−*^ mice have been generated and characterized previously^9,31^. For the pharmacological study WT mice (C57BL/6J, Jackson Laboratory) were used. Mice were held in specific-pathogen-free (SPF) condition, in a 12:12-h light–dark cycle, and with free access to purified food (E15430-047, Ssniff, Soest, Germany) and water. Experiments were approved by the Berlin Animal Review Board, Berlin, Germany (No. G0178/18) and followed the restrictions in the Berlin State Office for Health and Social Affairs (LaGeSo)^54^. All experiments were performed in accordance with ARRIVE guidelines^55^.

### Renal IRI model

Renal IRI was induced as described earlier^34^. Briefly, male mice (aged 14–18 weeks) were anaesthetized by isoflurane (2.3%) in air (350 ml/min). Preemptive analgesia with buprenorphine (0.2 mg/100 g) was used^56^. Mice were operated individually to ensure similar exposure to isoflurane^57^. Body temperature was maintained at 37 °C and monitored during surgery. Ischemia was induced after right–sided nephrectomy by clipping the pedicle of the left kidney for 17.5 or 20 min with a non–traumatic aneurysm clip (FE690K, Aesculap, Germany). Reperfusion was confirmed visually and the abdomen and the skin were sutured separately with a 5/0 braided-silk suture. After surgery mice had free access to water and chow. Body–warm sterile physiological saline solution (1 ml) was applied subcutaneously to every mouse. Sham operation was performed in a similar manner, except for clamping the renal pedicle. Twenty–four hours after reperfusion, mice were sacrificed by overdose of isoflurane and additionally cervical dislocation, and kidney and blood samples were collected for further analysis. The kidneys were divided into three portions. Upper pole of the kidney tissue was frozen and used for measurement of SH045 concentration. Middle part of kidney was immersed in 4% phosphate–buffered saline (PBS)–buffered formalin for histology, and the other left tissue was snap–frozen in liquid nitrogen for RNA preparation.

### TRPC6 blocker

Larixyl-6-N-methylcarbamate, also called SH045, is a compound with high affinity and subtype selectivity toward TRPC6 described by Häfner et al.^22^. SH045 was initially dissolved in DMSO (ratio of DMSO to vehicle is 0.5%) and then in 5% Cremophor EL^®^ solution with 0.9% NaCl and used for intravenous injection (2 mg/kg) 30 min before IRI surgery in the pharmacological studies with WT mice. BI-749327 is an orally bioavailable TRPC6 antagonist as reported^14^. BI-749327 (MedChemExpress, New Jersey, USA) was dissolved in DMSO and then suspended in corn oil (final ratio of DMSO to corn oil is 5%). For administration, 30 mg/kg of the final dose was delivered to mice via oral gavage 60 min before IRI surgery.

### Blood measurements and drugs

To allow repeated blood measurements of sodium, potassium, chloride, ionized calcium, total carbon dioxide, glucose, urea nitrogen, creatinine, hematocrit, hemoglobin and anion gap within a short time interval in the same mouse, 95 μL blood was taken from the facial vein and parameters were measured using an i-STAT system with Chem8 + cartridges (Abbott, USA).

### Quantitative real-time (qRT)-PCR

qRT-PCR was performed as described previously^9^. Briefly, Total RNA from snap-frozen kidneys were isolated using RNeasy RNA isolation kit (Qiagen, Australia) according to manufacturer’s instruction after homogenization with a Precellys 24 homogenizator (Peqlab, Germany). RNA concentration and quality were determined by NanoDrop-1000 spectrophotometer (Thermo Fisher Scientific, USA). Two micrograms of RNA were transcribed to cDNA (Applied Biosystems, USA). Quantitative analysis of target mRNA expression was performed with qRT-PCR using the relative standard curve method. TaqMan and SYBR green analysis was conducted using an Applied Biosystems 7500 Sequence Detector (Applied Biosystems, USA). The expression levels were normalized to 18S. Primer sequences are provided in Supplementary Table S1.

### Histology and analysis

Formalin-fixed, paraffin-embedded sections (2 μm) of kidneys were subjected to Periodic acid Schiff (PAS) stain according to the manufacturer’s protocols (Sigma, Germany). Semi-quantitative scoring of tubular damage was performed in a blinded manner in those 12 to 15 images at 200 × magnification per sample. Acute tubular injury (ATI) was observed in this study to assess the reversible tubular damage due to ischemia. The histologic features of ATI included one or more of the following lesions: tubular epithelial swelling with lucency of the cytoplasm, loss of brush border, luminal dilatation with simplification of the epithelium, and cytoplasmic vacuolization. Acute tubular necrosis (ATN) was also evaluated, which was indicated by patchy loss of tubular epithelial cells with resultant gaps and exposure of denuded basement membrane, evidence of cellular regeneration, as well as obliterated tubular hyaline and/or granular casts. The histological findings were graded from 0 to 3 according to the distribution of lesions: 0 = none; 1 =  < 25%; 2 = 25–50%; 3 =  > 50%^56^. The total score was calculated as sum of all morphological parameters. Tubular damage and tubular necrosis were quantified by an experienced renal pathologist who was unaware of Trpc6 genotypes or of treatment.

### HPLC and tandem mass spectrometric method (LC–MS/MS)

Concentration of SH045 in kidney homogenates was measured as previously described^23^. In summary, analyses were performed with an Agilent 1260 Infinity quaternary HPLC system (Agilent Technologies, Germany) consisting of a G4225A degasser, G1312B binary pump, G1367E autosampler, G1330B thermostat, G1316A column oven and G4212B diode array detector, coupled to a tandem QTRAP 5500 hybrid linear ion-trap triple quadrupole mass spectrometer (AB SCIEX, Canada). Data were acquired and processed using Analyst software (Version 1.7.1, AB SCIEX). Linear regressions and calculations were done using Multiquant software (Version 2.1.1, AB SCIEX). Lower limit of quantification (LLOQ) and accuracy were determined for quantification of SH045 effects in kidney and both revealed as high as reported for plasma by Chai et al.^38^.

### Immunofluorescence and analysis

We performed immunofluorescence similarly as previously described^34^. Two-μm thick sections of IRI-injured kidneys were post-fixed in ice-cold acetone, air-dried, rehydrated and blocked with 10% normal donkey serum (Jackson ImmunoResearch, America) for 30 min. Then sections were incubated in a humid chamber overnight at 4 °C with rat anti-Ly6B.2 (Gr1) (1:300; MCA771G; Bio-Rad AbD Serotec, Germany). The bound anti-Ly6B.2 antibody was visualized using Cy3-conjugated secondary antibody (1:500; Jackson ImmunoResearch, America) by incubating the sections for 1 h in a humid chamber at room temperature. Positive cells were counted in the outer medulla on ten non-overlapping view fields at 400 × magnification and mean cell numbers were taken for analysis.

### Isolated perfused kidneys

Isolated kidneys were perfused in an organ chamber using a peristaltic pump (Instech, USA) at constant flow (0.3–1.9 ml/min) of oxygenated (95% O_2_ and 5% CO_2_) physiological salt solution (PSS) containing (in mmol/L) 119 NaCl, 4.7 KCl, 1.2 KH_2_PO_4_, 25 NaHCO_3_, 1.2 Mg_2_SO_4_, 11.1 glucose, 1.6 CaCl_2_). Hyperforin (Sigma-Aldrich, USA), BI-749327, and SH045 were dissolved in DMSO. Before application, the aliquots were dissolved 1:1000 in PSS. So, the final concentration of DMSO did not exceed 0.1%. The concentrations of hyperforin, BI-749327, SH045 were 10 µM, 100 nM, and 100 nM, respectively. We used PSS as a solvent for Ang II (LKT Laboratories Inc., USA) and applied it at 10 nM concentration. Drugs were added to the perfusate. Perfusion pressure was detected using pressure transducer after an equilibration period of 60 min. Powerlab system (AD Instruments, Colorado Springs) was used for data acquisition and analysis. Ang II-induced pressor effects were normalized to the maximal pressor effect obtained with KCl (60 mmol/L)^58^. The composition of 60 mM KCl (in mmol/L) was 63.7 NaCl, 60 KCl, 1.2 KH_2_PO_4_, 25 NaHCO_3_, 1.2 Mg_2_SO_4_, 11.1 glucose, and 1.6 CaCl_2_.

### Statistics

Statistical analysis was performed using GraphPad 5.04 software. Study groups were analyzed by one-way ANOVA using Turkey’s post-hoc test or two-way ANOVA using Sidak’s multiple comparisons post hoc test. Data are presented as mean ± SEM. *P* values < 0.05 were considered as statistically significant.

### Experimental statement

All methods were carried out in accordance with relevant guidelines and regulations.

## Supplementary Information

Supplementary Information 1.

Supplementary Information 2.

Supplementary Information 3.

Supplementary Information 4.

Supplementary Information 5.

Supplementary Information 6.

Supplementary Information 7.

Supplementary Information 8.

Supplementary Information 9.
