# Supplementary figures and images for: Role of TRPC6 in kidney damage after acute ischemic kidney injury
Source: Sci Rep. 2022 Feb 22;12:3038. doi: 10.1038/s41598-022-06703-9 (PMC8864023; doi:10.1038/s41598-022-06703-9)

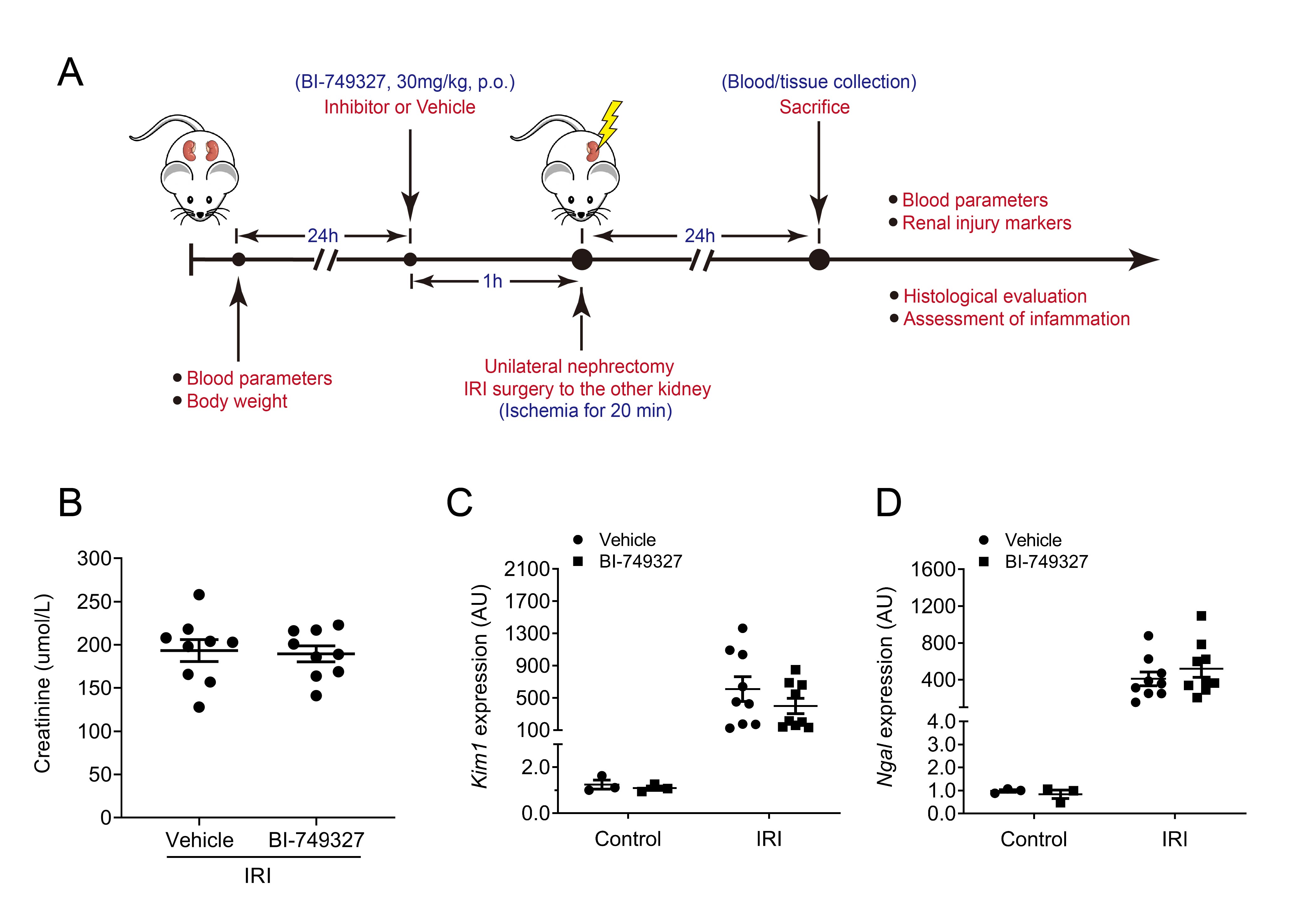

Supplement: Supplementary file 1 — Supplementary Information 1. [file 41598_2022_6703_MOESM1_ESM.jpg]

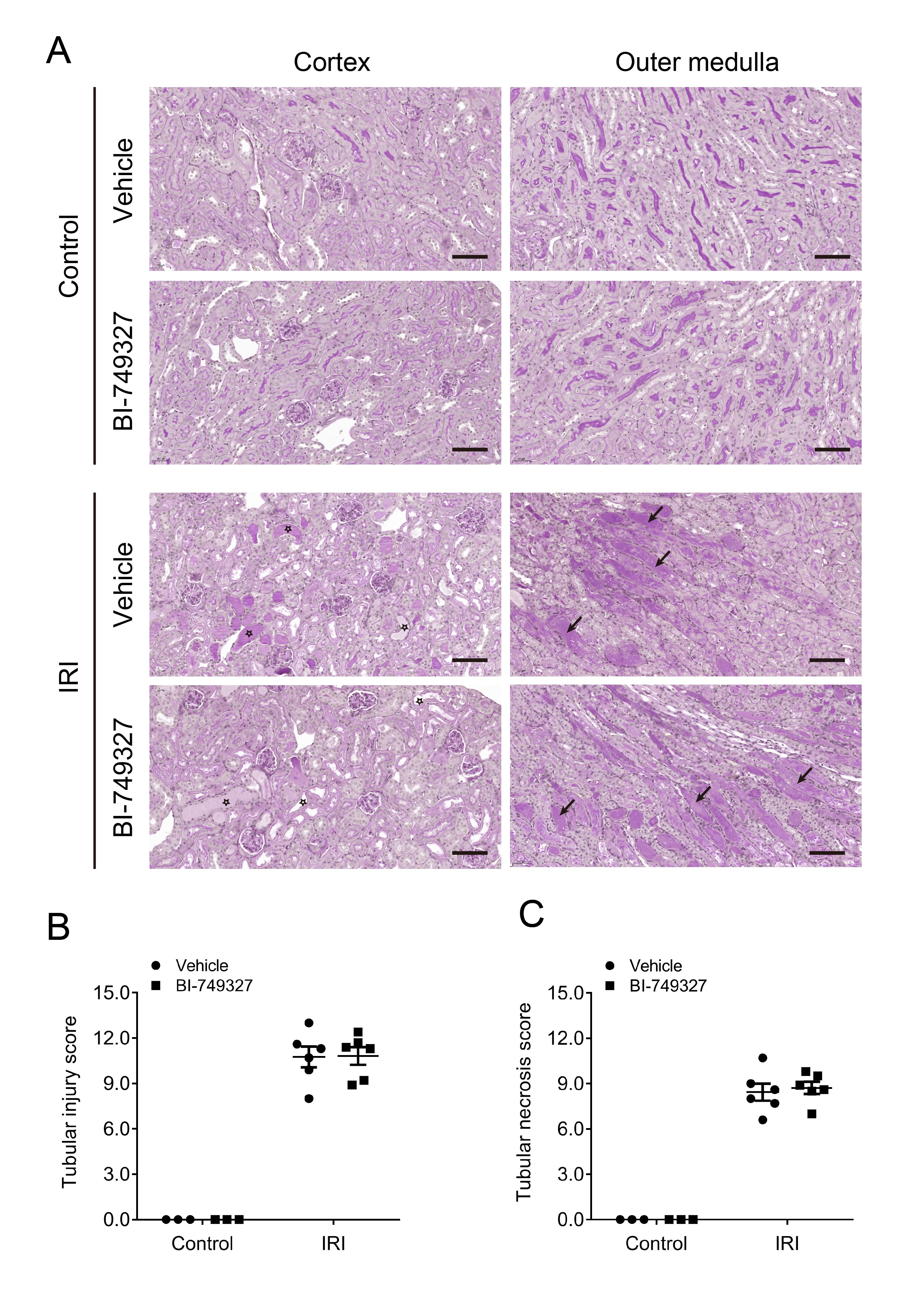

Supplement: Supplementary file 2 — Supplementary Information 2. [file 41598_2022_6703_MOESM2_ESM.jpg]

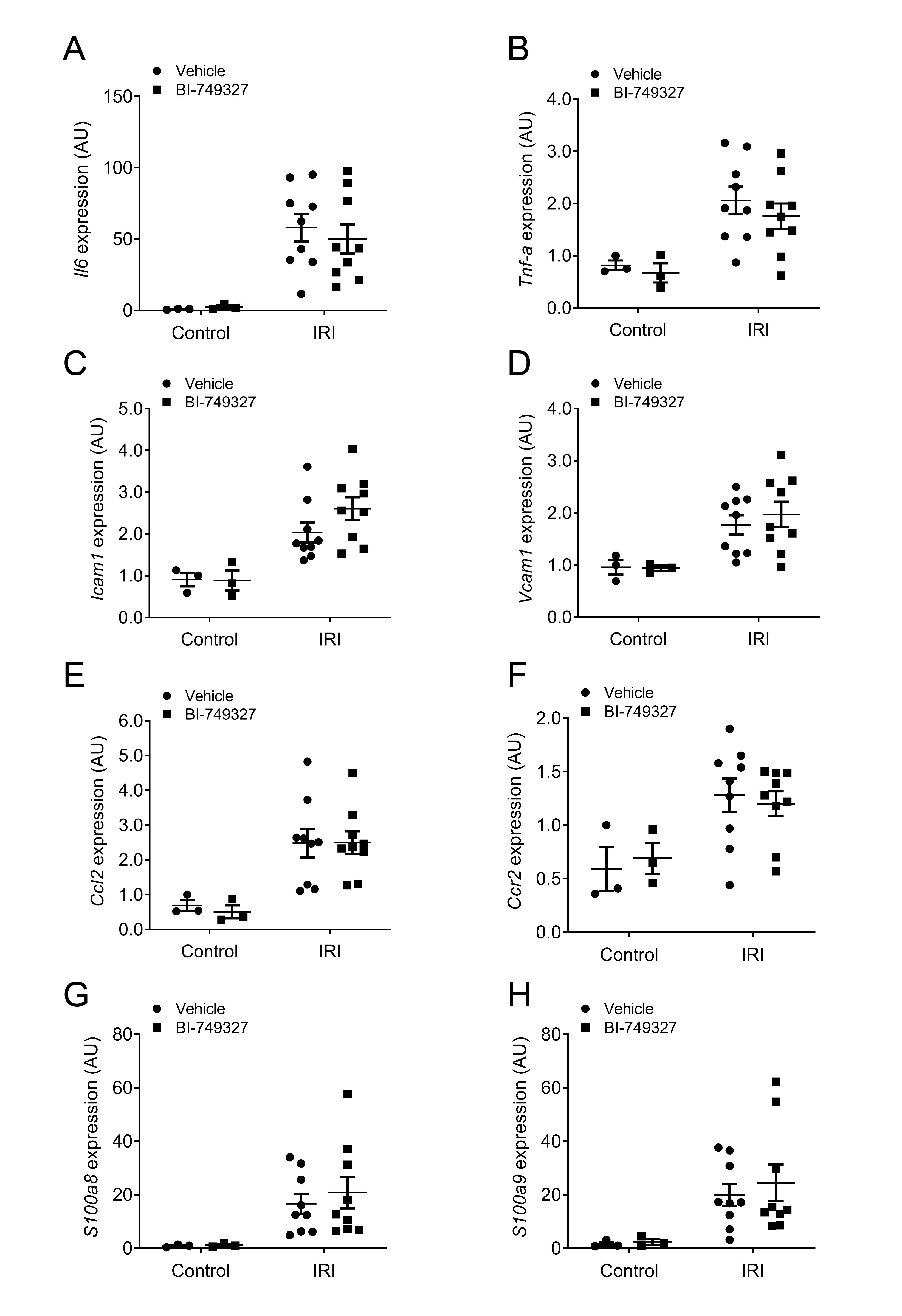

Supplement: Supplementary file 3 — Supplementary Information 3. [file 41598_2022_6703_MOESM3_ESM.jpg]

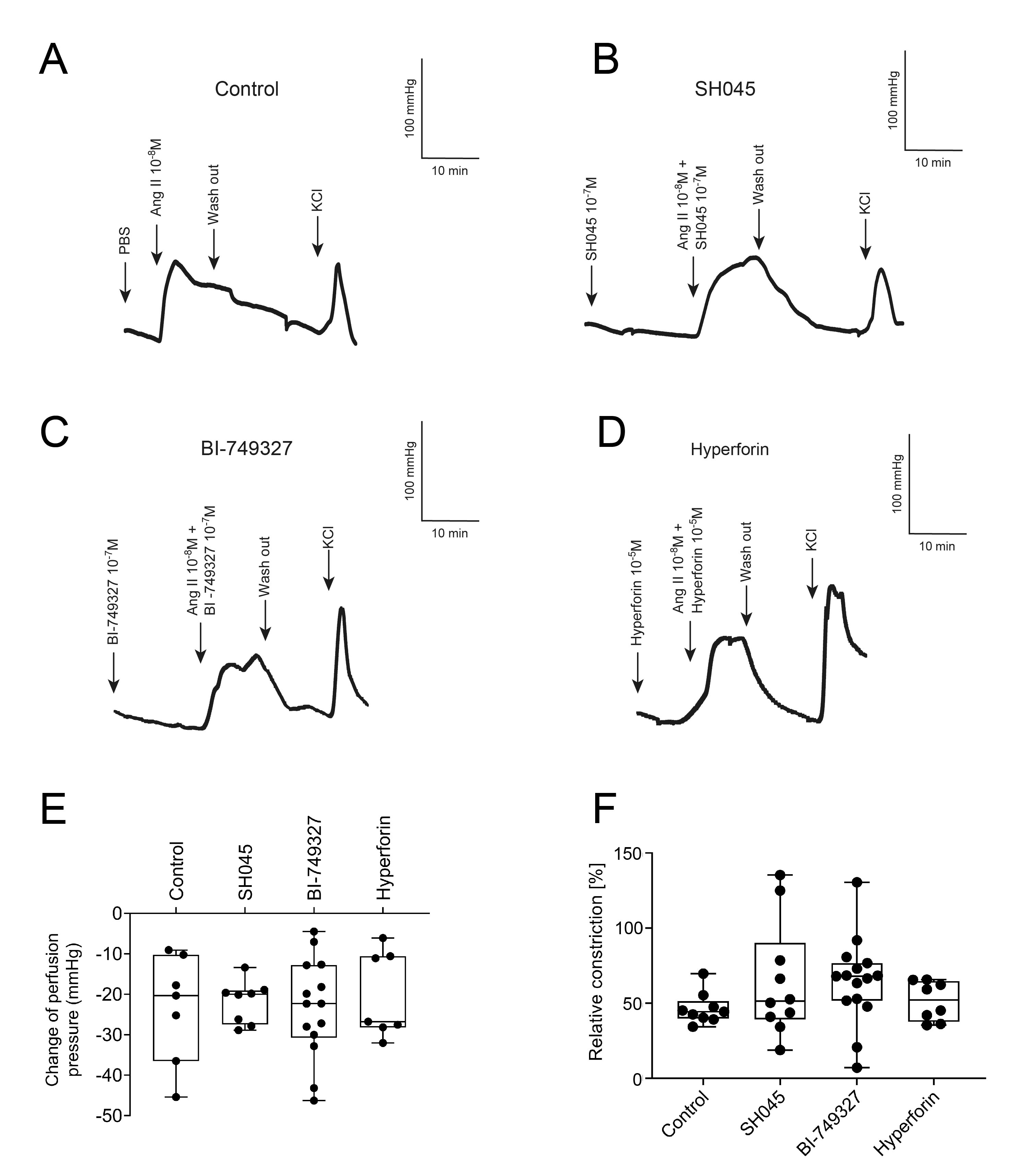

Supplement: Supplementary file 4 — Supplementary Information 4. [file 41598_2022_6703_MOESM4_ESM.jpg]

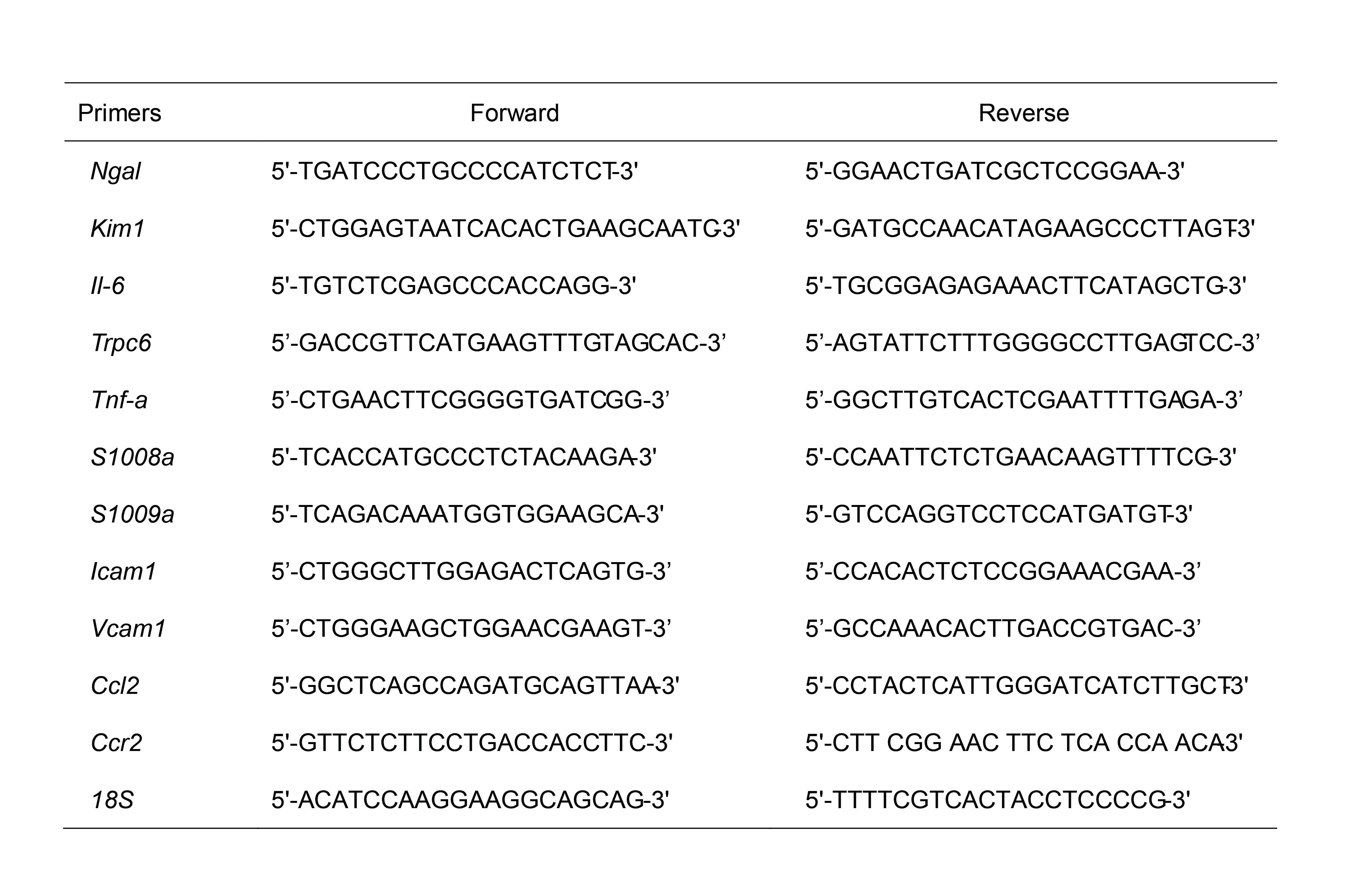

Supplement: Supplementary file 6 — Supplementary Information 6. [file 41598_2022_6703_MOESM6_ESM.jpg]

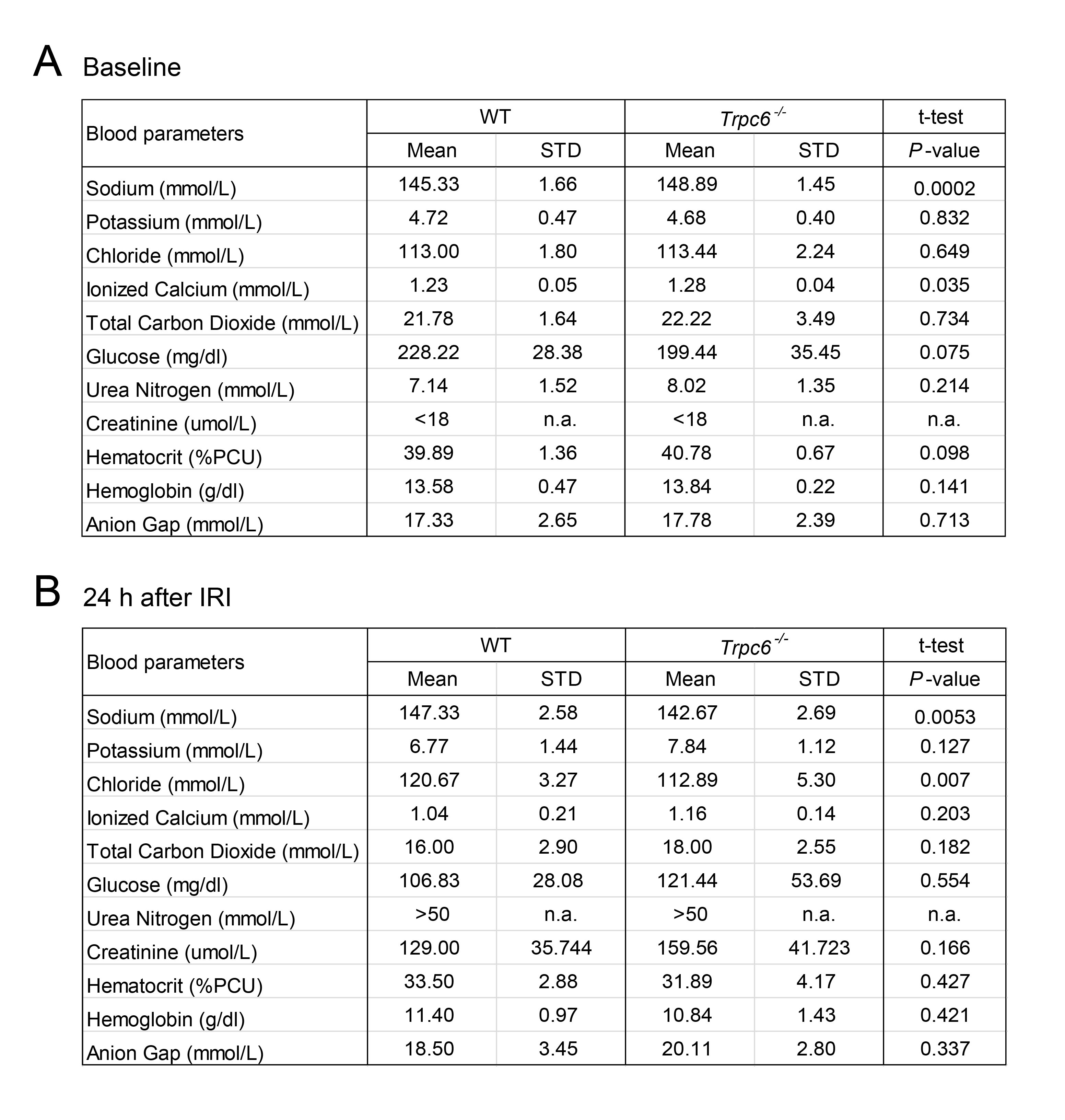

Supplement: Supplementary file 7 — Supplementary Information 7. [file 41598_2022_6703_MOESM7_ESM.jpg]

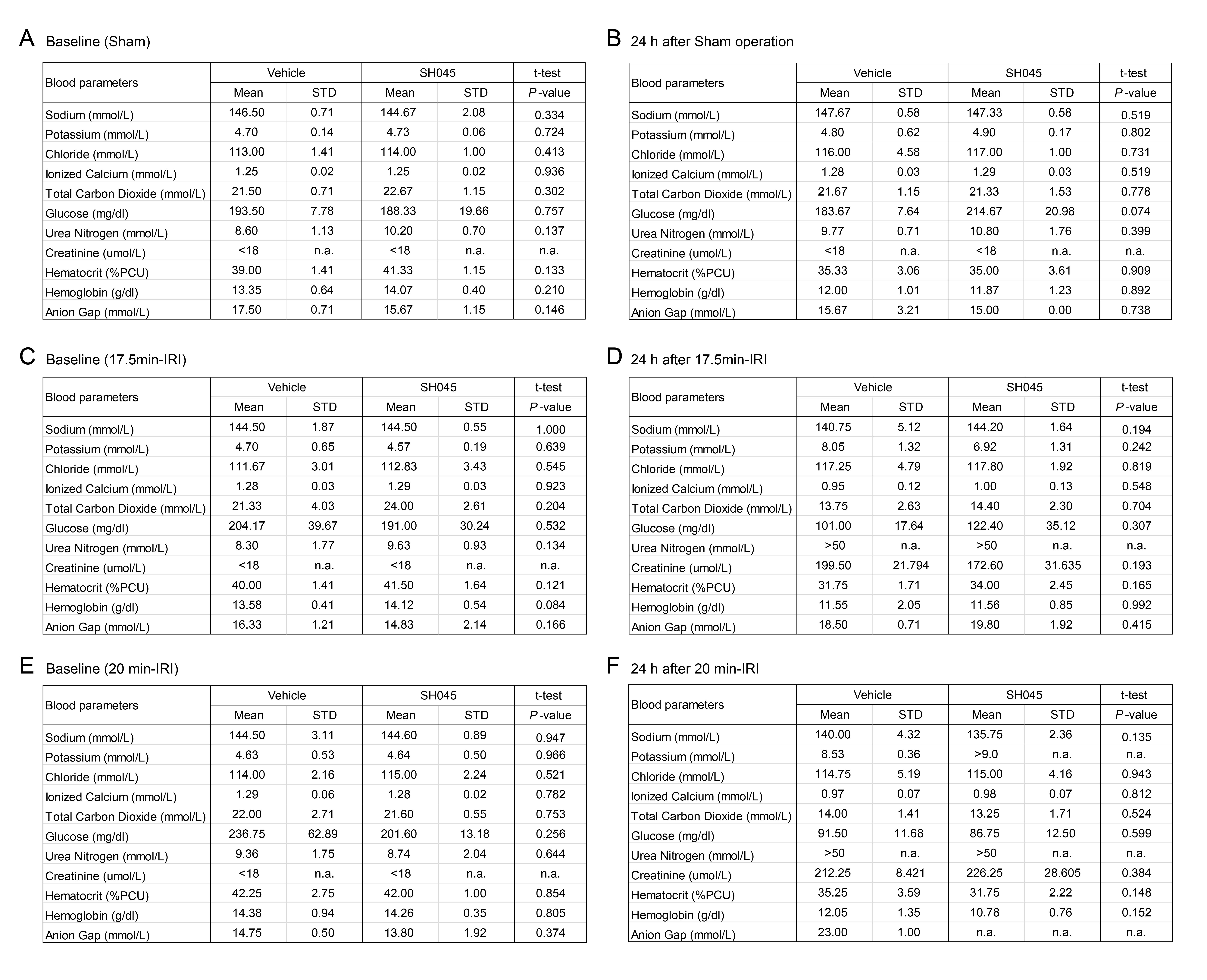

Supplement: Supplementary file 8 — Supplementary Information 8. [file 41598_2022_6703_MOESM8_ESM.jpg]

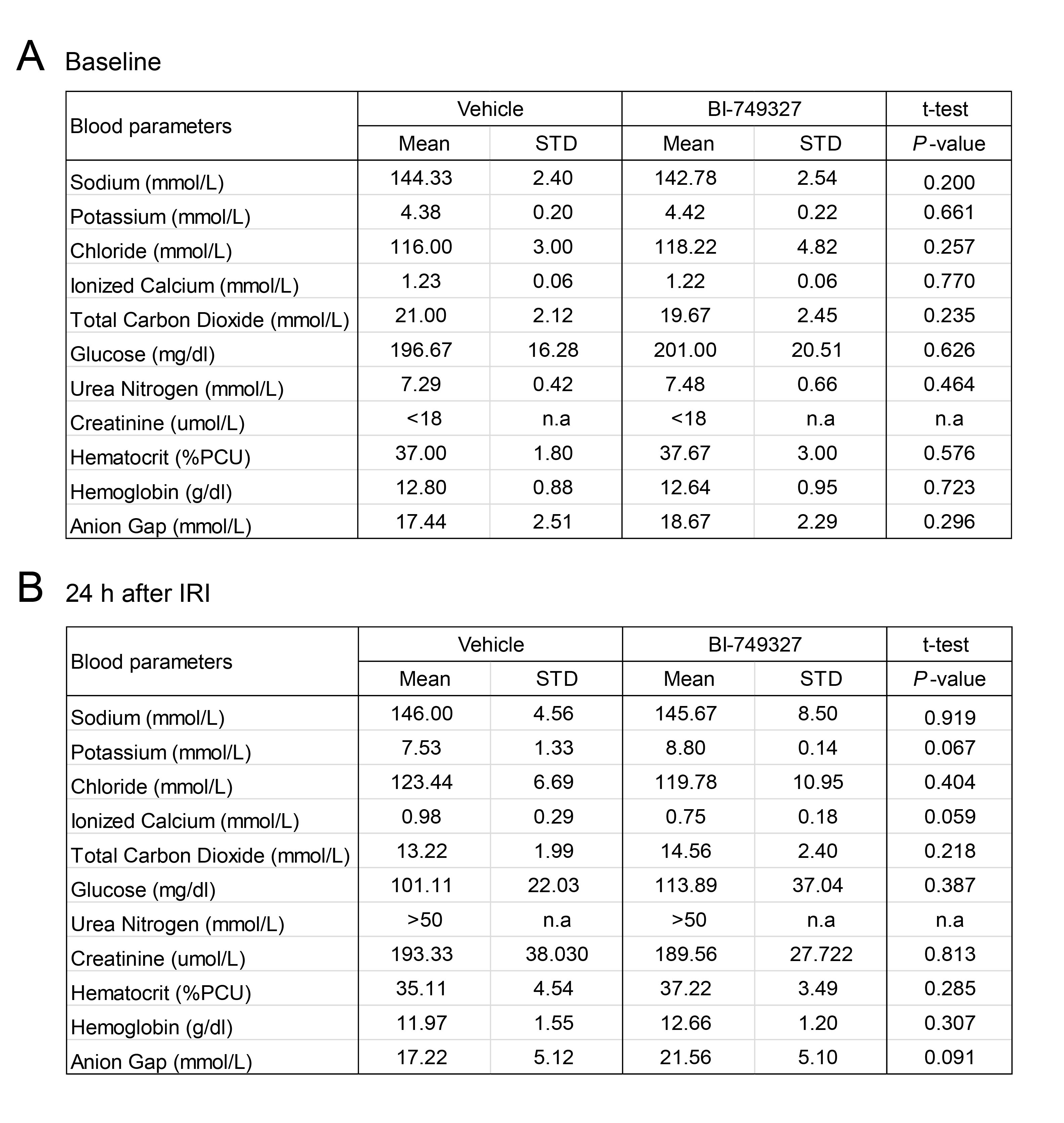

Supplement: Supplementary file 9 — Supplementary Information 9. [file 41598_2022_6703_MOESM9_ESM.jpg]
