# Supplementary material for: Role of TRPC6 in kidney damage after acute ischemic kidney injury
Source: Sci Rep. 2022 Feb 22;12:3038. doi: 10.1038/s41598-022-06703-9 (PMC8864023; doi:10.1038/s41598-022-06703-9)
Supplement: Supplementary file 5 — Supplementary Information 5. [file 41598_2022_6703_MOESM5_ESM.pdf]

Role of TRPC6 in Kidney Damage after Acute Ischemic Kidney Injury

Zhihuang Zheng[1,2,3], Dmitry Tsvetkov[1,2,4*], Theda Bartolomaeus[2,12], Cem Erdogan[5], Ute Krügel[6], Johanna Schleifenbaum[5], Michael Schaefer[6], Bernd Nürnberg[7], Xiaoning Chai[6], Friedrich-Alexander Ludwig[8], Gabriele N'diaye[2,12], May-Britt Köhler[2,12], Kaiyin Wu[9], Maik Gollasch[1,2,4*], Lajos Markó[2,10,11,12*]

Supplementary Information

Supplementary Figure S1. Effect of BI-749327 on renal function and renal damage markers. (A) Experimental design. (B) Serum creatinine levels (n=9 per group). (C) Renal expression of kidney injury molecule 1 (*Kim1*) and (D) neutrophil gelatinase-associated lipocalin (*Ngal*). Statistical testing was two-way ANOVA followed by Sidak's multiple comparisons post hoc test.

Supplementary Figure S2. Effect of BI-749327 on kidney histopathology after AKI. (A) Representative images from the cortico-medullar region of control and IRI-injured kidneys of vehicle or BI-749327-treated mice (magnification: 200×). Kidney sections are Periodic Acid-Schiff (PAS) stained. Arrows indicate tubular necrosis. Stars indicate tubular injury. Scale bars are 100 µm. (B) Semi-quantification of tubular injury. (C) Semi-quantification of tubular necrosis. Data expressed as mean ± SEM (Control n=3 each, and IRI n=6 each, respectively). Statistical testing was performed using two-way ANOVA followed by Sidak's multiple comparisons post hoc test.

Supplementary Figure S3. Effect of BI-749327 on renal gene expression of inflammatory markers. (A) Renal expression of interleukin 6 (*Il6*) and (B) tumor necrosis factor-α (*Tnf-α*), (C) intercellular adhesion molecule 1 (*Icam1*), (D) vascular cell adhesion protein 1 (*Vcam1*), (E) C-C motif chemokine 2 (*Ccl2*), (F) C-C motif chemokine receptor 2 (*Ccr2*), and (G and H) S100 calcium-binding protein A8/9 (*S100a8/9*) (Control n=3 each, and IRI n=9 each, respectively). Data expressed as mean ± SEM. Statistical testing was performed using two-way ANOVA followed by Sidak's multiple comparisons post hoc test. AU, arbitrary units.

Supplementary Figure S4. Vasoregulation in isolated perfused kidneys. (A) Original recordings of perfusion pressure in kidneys perfused with PSS (control), (B) TRPC6 blocker SH045, (C) another TRPC6 blocker BI-749327, and (D) TRPC6 agonist hyperforin. (E) Decrease of perfusion pressure (n = 7, 8, 14, 7 for control, SH045, BI-749327, and hyperforin, respectively). (F) Increase in perfusion pressure induced by 10 nM Ang II normalized to 60 mM KCl (n = 9, 10, 15, 8 for Control, SH045, BI-749327, and hyperforin, respectively). One-way ANOVA followed by Dunnett's multiple comparisons test.

Supplementary Table S1. The sequences of murine gene primer.

Supplementary Table S2. (A) Baseline serum parameters of WT or *Trpc6*$^{-/-}$ mice before IRI surgery (n=9 per group). (B) Serum parameters of sham WT or *Trpc6*$^{-/-}$ mice at 24 hours after IRI surgery (n=7, 9 for IRI WT and *Trpc6*$^{-/-}$, respectively). Data expressed as means ± STD. Two-tailed unpaired t-test. n.a. = not applicable.

Supplementary Table S3. (A) Baseline serum parameters of sham mice before surgery (n=3 per group). (B) Serum parameters of sham mice after surgery (n=3 per group). (C) Baseline serum parameters of 17.5 min-IRI mice before surgery (n=5 per group). (D) Serum parameters of 17.5 min-IRI mice after surgery (n=4, 5 for 17.5 min-IRI in vehicle and SH045, respectively). (E) Serum parameters of 20 min-IRI mice before surgery (n=4 per group). (F) (E) Serum parameters of 20 min-IRI mice after surgery (n=4 per group). Data expressed as means ± STD. Two-tailed unpaired t-test. n.a. = not applicable.

Supplementary Table S4. (A) Baseline serum parameters of sham mice before surgery (n=9 per group). (B) Serum parameters of IRI mice after surgery (n=9 per group). Data expressed as means ± STD. Two-tailed unpaired t-test. n.a. = not applicable.
